# Flumazenil-Insensitive Benzodiazepine Effects in Recombinant αβ and Neuronal GABA_A_ Receptors

**DOI:** 10.3390/brainsci10030150

**Published:** 2020-03-05

**Authors:** Jing-Jing Lian, Yan-Qing Cao, Yu-Lei Li, Gang Yu, Rui-Bin Su

**Affiliations:** State Key Laboratory of Toxicology and Medical Countermeasures, Beijing Key Laboratory of Neuropsychopharmacology, Beijing Institute of Pharmacology and Toxicology, 27th Taiping Road, Beijing 100850, China; alianjingjing@163.com (J.-J.L.); cyqorange@163.com (Y.-Q.C.); bettylpaine@126.com (Y.-L.L.); ruibinsu@126.com (R.-B.S.)

**Keywords:** diazepam, benzodiazepine, GABA_A_ receptors, αβ receptors, benzodiazepine binding sites

## Abstract

Gamma-aminobutyric acid, type A (GABA_A_) receptors are complex heterogeneous pentamers with various drug binding sites. Several lines of evidence suggest that benzodiazepines modulate certain GABA_A_ receptors in a flumazenil-insensitive manner, possibly via binding sites other than the classical ones. However, GABA_A_ receptor subtypes that contain non-classical benzodiazepine binding sites are not systemically studied. The present study investigated the high-concentration effects of three benzodiazepines and their sensitivity to flumazenil on different recombinant (α1β2, α2β2, α3β2, α4β2, α5β2 and α1β3) and native neuronal GABA_A_ receptors using the whole-cell patch-clamp electrophysiology technique. The classical benzodiazepine diazepam (200 μmol/L) and midazolam (200 μmol/L) produced flumazenil-insensitive effects on α1β2 receptor, whereas the imidazopyridine zolpidem failed to modulate the receptor. Flumazenil-insensitive effects of diazepam were also observed on the α2β2, α3β2 and α5β2, but not α4β2 receptors. Unlike β2-containing receptors, the α1β3 receptor was insensitive to diazepam. Moreover, the diazepam (200 μmol/L) effects on some cortical neurons could not be fully antagonized by flumazenil (200 μmol/L). These findings suggested that the non-classical (flumazenil-insensitive) benzodiazepine effects depended on certain receptor subtypes and benzodiazepine structures and may be important for designing of subtype- or binding site- specific drugs.

## 1. Introduction

Gamma-aminobutyric acid, type A (GABA_A_) receptors are ligand-gated chloride channel receptors that primarily mediate inhibitory neurotransmission in the central nervous system [1,2]. GABA_A_ receptors are complex heterogeneous pentamers that made up from at least 19 different subunits (including α1–6, β1–3, γ1–3, δ, ε, θ, π, and ρ1–3) with distinct regional, cellular and subcellular distribution in mammalian brain [3]. The diversity of the subunits, their distribution characteristics and different assembly patterns give rise to various GABA_A_ receptor subtypes with distinct physiological and pharmacological properties, which are involved in the modulation of distinct functions of the brain [3,4,5,6]. The majority of GABA_A_ receptors were considered to be composed of two α, two β and one γ (or δ) subunits [1,6,7]. However, the existence and functional properties of αβ receptors (GABA_A_ receptor subtypes containing only α and β subunits) in the central nervous system has been recognized [4,8,9]. These receptors sensed relatively low ambient GABA concentrations and seemed to exclusively locate extrasynaptically exerting tonic inhibition [8], which plays important roles in various networks [9].

Benzodiazepines, which are positive allosteric modulators of GABA_A_ receptors, are among the most commonly prescribed drugs with versatile pharmacological actions, including anxiolysis, anti-convulsion, sedation, hypnosis and muscle relaxation [10,11,12]. The interaction of benzodiazepine and GABA_A_ receptors have been extensively investigated but far from clarified. A specific benzodiazepine binding site (classical binding site) has been well-recognized to be located at the extracellular αx+/γy− interfaces of the GABA_A_ receptors (x = 1,2,3,5; y = 1–3) [13]. Several non-classical benzodiazepine binding sites have been subsequently revealed on certain GABA_A_ receptor subtypes [14,15], and benzodiazepine effects via non-classical binding sites were insensitive to flumazenil, a specific benzodiazepine antagonist at classical binding sites [15]. Additionally, our previous study showed evidence of non-classical binding sites in vivo [16]. Moreover, benzodiazepines modulate α1β2 [17,18] and α1β3 [19,20,21] receptors and these two receptor subtypes were used as experimental models to study certain non-classical benzodiazepine binding sites [17,20]. However, the non-classical benzodiazepine effects are not systemically studied in αβ receptor subtypes, and the contribution of the subunits in forming the non-classical binding sites are unclear.

The present study investigated the flumazenil-insensitive benzodiazepine effects on a series of recombinant αβ receptors in HEK293 cells using the whole-cell patch-clamp technique, suggesting that flumazenil-insensitive effects depend on both the receptor subtypes and the benzodiazepine structures. Furthermore, the non-classical benzodiazepine effects were observed on some cortical neurons. These findings are important for elucidating the mechanisms of benzodiazepines, and may be valuable for developing novel drugs.

## 2. Materials and Methods

### 2.1. Chemicals

GABA and CsCl were obtained from Sigma-Aldrich (St. Louis, MO, USA). Cs_4_BAPTA, CsF were purchased from Tocris (Bristol, UK) and HEPES from Amresco (Boise, IA, USA). NaCl, KCl, MgCl_2_ and CaCl_2_ were bought from Sinopharm Chemical Reagent Company (Beijing, China). Zolpidem tartrate and flumazenil were purchased from National Institutes for Food and Drug Control (Beijing, China). Diazepam and midazolam were obtained from Jiangsu Enhua Pharmaceutical Co., Ltd. (Xuzhou, China).

Stored solutions of diazepam, midazolam, zolpidem tatrate and flumazenil at 100, 100, 50 and 100 mmol/L were made in dimethyl sulfoxide (DMSO) and stored at −20 °C. All drugs were diluted to the needed concentrations using extracellular solution (as described in Section 2.5) at room temperature on the day of experiment. GABA was dissolved in extracellular solution at the required concentrations on the day of experiment.

### 2.2. Expression of GABA_A_ Receptors in HEK293 Cells

cDNAs of rat GABA_A_ receptor α1, α2, α3, α4, α5, β2 and β3 subunits were obtained from rat brain mRNA by reverse transcription PCR. cDNAs of α1, α2, α3, α4 and α5 subunits were cloned into vector pcDNA 3.1 (+) (Sigma, St. Louis, MO, USA) and that of β2 and β3 subunits into vector pIRES2 (EGFP) (Clontech, Mountain View, CA, USA), which allowed the expression of both the β2 or β3 subunit and green fluorescent protein from an internal ribosomal entry site (IRES). Plasmid Mini Kit (Omega, Doraville, GA, USA) was used to purify plasmids. HEK293 cells (ATCC, Manassas, VA, USA) were transfected with combinations of different α and β/EGFP subunits cDNAs using Lipofectamine 3000 transfection kit (Invitrogen, Carlsbad, CA, USA). The transfection mixture contained 1 μg α and 1 μg β/EGFP. The cells were propagated and plated on poly-l-lysine-coated glass coverslips in 35 mm dishes 10 h after transfection. EGFP-positive cells were used for electrophysiological recording 48 h after transfection.

### 2.3. Primary Cortex and Hippocampus Neuronal Cultures

Cortical and hippocampal neurons were prepared from cortex and hippocampus of neonatal (P0,P1) Sprague-Dawley rats. Rats were decapitated and the extracted brains were placed in a dish containing ice-cold solution containing (in mmol/L): 136.75 NaCl, 2.68 KCl, 8.10 Na_2_HPO_4_, 1.47 KH_2_PO_4_, 16.65 glucose and 21.91 sucrose. The cortex and hippocampus were dissected from brains under a microscope, blood vessels and meninges were removed subsequently. Cortical and hippocampal tissues were transferred to two distinct 10 mL centrifuge tube, chopped and dissociated with 0.25 mg/mL trypsin at 37 °C for 15 min. Seeding medium (DMEM medium supplemented with 10% fetal calf serum and 10% equine serum) was then added into the tissues to terminate dissociation. Centrifuge the tissues at 1500 rpm for 5 min to filter the cells. The cells were dispersed in seeding culture and plated in 35 mm dishes, containing two glass coverslips coated with poly L-lysine hydrobromide (0.1 mg/mL), at a density of 1 × 10^6^ cells/mL. Twenty-four hours later, the seeding medium was replaced with the culture medium (Neurobasal medium with 2% B27 supplement and penicillin-streptomycin). On the fifth day of plating, Cytosine arabinofuranoside (4 μmol/L, Sigma, St. Louis, MO, USA) was added to the culture medium to inhibit glial proliferation. Immunofluorescence was used to identify neurons (expressed in Section 2.4). The cells were cultured for 7–14 days prior to use. The experimental procedures were approved by the local ethical committee and the Institutional Review Committee on Animal Care and Use (IACUC of AMMS-06-2017-003).

### 2.4. Immunofluorescence

Neurons were fixed in ice-cold methanol for 10 min and then blocked in 5% bovine serum albumin solution in PBS for 1 h. The cells were permeabilized with 0.2% Triton X-100 in 0.2% BSA solution (pH 7.4) for 10 min and incubated with anti-160 kD neurofilament medium antibody (ab134458, Abcam, 1:100) for 2 h at room temperature. After three washes in PBS solution, neurons were incubated with Alexa 488-conjugated anti-goat secondary antibody (ab150113, Abcam, 1:500) in the dark at room temperature for 1 h. Neurons were then visualized using the laser-scanning microscope (Zeiss LSM 880 Pascal) and the images were acquired with a digital camera.

### 2.5. Whole-Cell Patch-Clamp Electrophysiology

GABA-gated currents were recorded on recombinant αβ receptors or cortical and hippocampal neurons in the whole-cell voltage-clamp mode using the 700B Axopatch amplifier (Axon Instruments) at a holding potential of −60 mV. Patch pipettes (7–10 MΩ for HEK293 cells and 3–6 MΩ for neurons) were pulled from borosilicate glass capillaries and filled with internal electrode solution containing (in mmol/L): CaCl_2_ 0.5, MgCl_2_ 1, Cs_4_BAPTA 5, CsF 10, HEPES 10, CsCl 135; pH 7.4. The cells were perfused in extracellular solution consisted of (in mmol/L): NaCl 135, KCl 5, HEPES 10, MgCl_2_ 1, CaCl_2_ 1.8; pH 7.4. In recording of neurons, the bath solution was supplemented with tetrodotoxin (0.5 μmol/L). Currents were filtered at 10 kHz and digitized using Digidata 1550B (Axon Instruments), pClamp10 software (Axon Instruments) was used for data (peak amplitude) acquisition. GABA or benzodiazepines at different concentrations were applied to the patched cells through manually controlled pulses (3–6 s) [22] using a micropipette coupled to a microsyringe. The diameter of the application pipette tip was 100 µm and it was placed 300–400 µm from the patched cell. Meanwhile, a flow system (ALA, USA) was used to perfuse extracellular solution during the experiment to wash out drugs, so as to avoid desensitization.

To test whether co-transfection of cDNAs of different α and β subunits (in Section 2.2) yielded GABA gated receptor channels, currents elicited by increasing GABA concentrations were recorded for different recombinant receptors. Enhancements of GABA-induced currents by benzodiazepines were measured by co-applying of benzodiazepines and GABA. GABA concentrations that elicited about 3–10% of the maximal current amplitude (EC_3–10_) were adopted as determined at the beginning of each experiment. Diazepam at micromolar concentrations (20–150 μmol/L) was reported to modulate the recombinant α1β2 receptor in oocytes [17]. 200 μmol/L diazepam was revealed to potentiate GABA-gated currents on α1β2 receptor transfected in HEK293 cells in our preliminary experiments. Thus, diazepam (200 μmol/L) was used in the present study. To avoid desensitization of the receptors, a washout period of 5 min was executed between drug applications. HEK293 cells transfected with cDNA of the α1, α2, α3, α4, α5, β2 or β3 subunit only did not result in functional expression.

### 2.6. Statistical Analysis

The pClamp10 software (Axon Instruments) was used to quantify peak amplitude. GraphPad Prism 5.0 (GraphPad Software Inc, La Jolla, CA, USA) was used for all statistical analysis. GABA effects were normalized to maximal responses. Non-Linear regression was performed among GABA concentrations and the electrophysiological effects, the concentration–response was fitted with a sigmoidal curve using Hill-function according to equation (1) (provided by GraphPad), so as to estimate the half maximal effective concentration (EC_50_). Differences between distinct groups were analyzed by one-way analysis of variance (ANOVA) with Dunnett’s post hoc tests. Values are expressed as means ± standard error (SEM) and the criterion for statistically significant difference was the value of *p* less than 0.05.

*I = I_max_*/(1 + 10^[(LogEC_50_ − X) * H]) (1)

where *I* represents the current and *I_max_* is the maximum current, X is the Log agonist concentration and H represents the Hill Coefficient.

## 3. Results

### 3.1. GABA Concentration-Dependent Activation of Recombinant αβ Receptors

**Figure 1:**
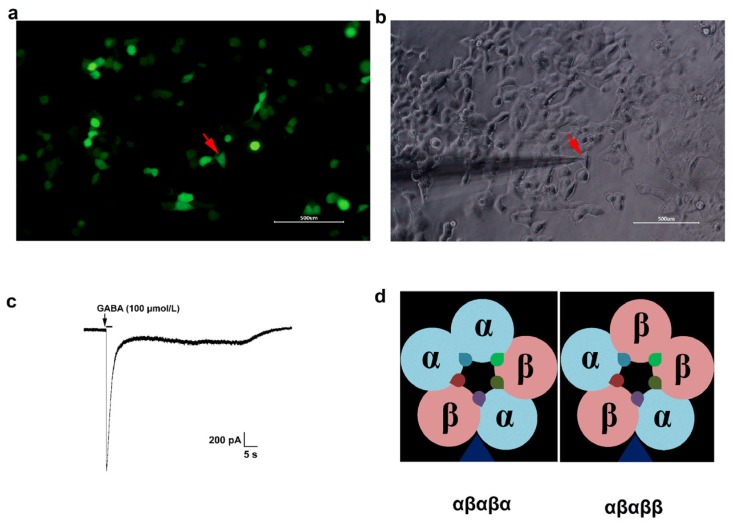
Expression of αβ receptors in HEK293 cells. HEK293 cells were co-transfected with cDNAs of different α and β/EGFP in a ratio of 1:1, cells showed green fluoresce 48 h after transfection (**a**). EGFP-positive cells were subjected to whole-cell voltage-clamp electrophysiology with a holding potential of −60 mV, red arrowheads indicated the patched cell (**b**). GABA evoked currents when applied to the patched cell (**c**). (**d**) α and β subunits can assembled in two stoichiometries of three α subunits with two β subunits (αβαβα) or two α subunits with three β subunits (αβαββ), 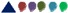 represents the possible benzodiazepine binding sites.

**Figure 2:**
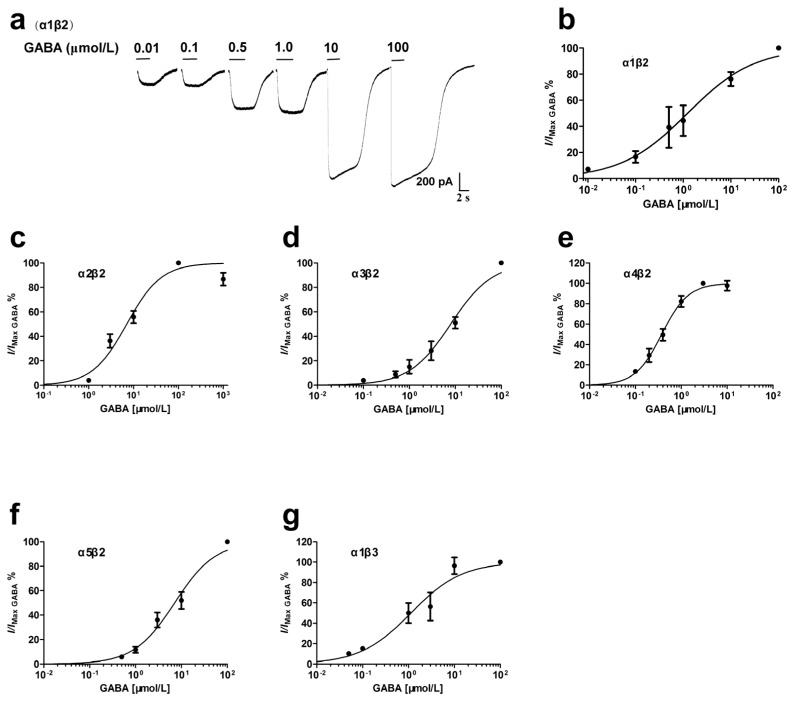
GABA concentration response relationships at recombinant αβ receptors. Effects of increasing concentrations of GABA (0.01–100 μmol/L) were estimated on different αβ receptors. Non-Linear regression was performed among GABA concentrations and electrophysiological effects and the concentration–response was fitted with a sigmoidal curve to estimate the half maximal effective concentrations (EC_50_). Each point represents the normalized peak currents from 3–5 cells. (**a**) The current traces from a single cell showing the various concentrations of GABA on α1β2 receptor. (**b**–**g**) The GABA concentration response curves on α1β2 (**b**), α2β2 (**c**), α3β2 (**d**), α4β2 (**e**), α5β2 (**f**) and α1β3 (**g**) receptors.

Cells showed green fluorescence, which indicates successful expression, 48 h after transfection (Figure 1a) and GABA (100 μmol/L) evoked currents when applied to the patched EGFP-positive cell (Figure 1c). GABA concentration-dependently activated α1β2 receptor and 100 μmol/L GABA produced the maximum effect (Figure 2a,b). The EC_50_ value was 1.23 μmol/L (Table 1), which was similar to the results from α1β2 receptor expressed in xenopus oocytes [17]. The α2β2, α3β2, α4β2, α5β2 and α1β3 receptors were also activated by GABA in a concentration-dependent manner, with EC_50_ values of 7.05, 7.94, 0.37, 7.12 and 1.13 μmol/L (Figure 2c–g, Table 1), which were similar to those estimated in previous studies [19,23,24,25,26]. Hill coefficient values were different among distinct receptors (Table 1). According to the EC_50_ values (Table 1), the relative order of GABA potency on αβ receptors was: α4β2 > α1β2 ≈ α1β3 > α3β2 > α5β2 ≈ α2β2.

### 3.2. α1β2 Receptor Showed Distinct Sensitivities to Different Benzodiazepines

**Figure 3:**
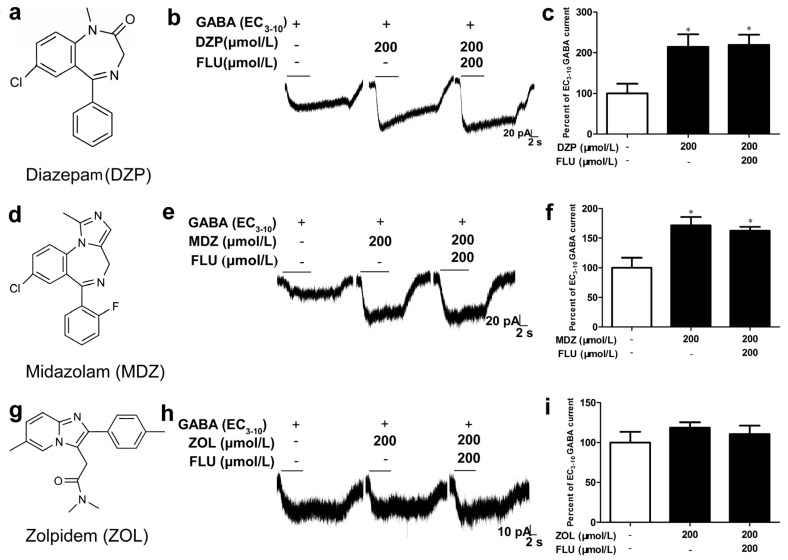
Sensitivity of α1β2 receptor to different benzodiazepines (BZDs). Effects of diazepam (DZP, **a**–**c**), midazolam (MDZ, **d**–**f**) and zolpidem (ZOL, **g**–**i**) in potentiating the GABA-evoked currents and their sensitivity to flumazenil (FLU) were estimated by co-applying of GABA (EC_3–10_, 0.1 μmol/L) and different BZDs or BZDs/FLU. (**a**,**d**,**g**) The chemical structure of DZP, MDZ and ZOL. (**b**,**e**,**h**) The current traces from cells with the treatment of DZP, MDZ and ZOL. One-way analysis of variance (ANOVA) with Dunnett’s post hoc tests; * *p* < 0.05, vs. control group; *n* = 4–5.

The effects of diazepam, midazolam and zolpidem, three classical benzodiazepine ligands with distinct chemical structures, were evaluated on the α1β2 receptor. The prototype l,4-benzodiazepine diazepam (200 μmol/L) (Figure 3a) elicited a marked modulatory effect on α1β2 receptor, potentiating the EC_3–10_ GABA current to 214.0 ± 31.4% (*p* < 0.05, *n* = 4), and the potentiation was not affected by 200 μmol/L flumazenil (Figure 3b,c). Similar to diazepam, midazolam (200 μmol/L), which owns the 1,2-annelated imidazo-benzodiazepine structure (Figure 3d), also significantly potentiated GABA current (171.8 ± 13.4%, *p* < 0.05, *n* = 4) in a flumazenil-insensitive manner (Figure 3e,f). However, the imidazopyridine zolpidem (200 μmol/L) (Figure 3g) failed to effectively modulate the receptor (Figure 3h,i)

### 3.3. The Contribution of α and β Subunits to the Sensitivity of αβ Receptor to Diazepam

**Figure 4:**
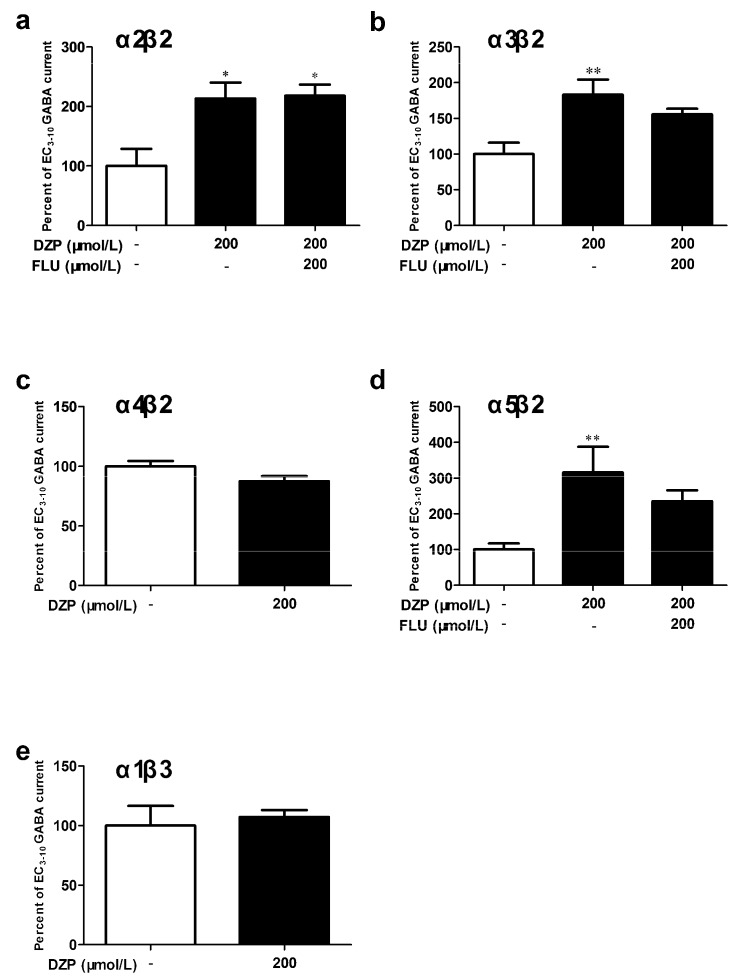
Modulatory effects of diazepam on different αβ receptors. GABA (EC_3–10_, 1 μmol/L for α2β2, 0.5 μmol/L for α3β2, 0.01 μmol/L for α4β2, 0.5 μmol/L for α5β2 and 0.1 μmol/L for α1β3) or a mixture of GABA/DZP or GABA/DZP/FLU were applied to α2β2 (**a**), α3β2 (**b**), α4β2 (**c**), α5β2 (**d**) and α1β3 (**e**) receptors. One-way ANOVA with Dunnett’s post hoc tests; * *p* < 0.05, ** *p* < 0.01 vs. control group; *n* = 3–6.

To clarify the contribution of specific α subunit in mediating benzodiazepine effect, the effects of diazepam on α2β2, α3β2, α4β2 and α5β2 receptors were tested. In concordance with the results on α1β2 (Figure 3b,c) receptor, diazepam (200 μmol/L) significantly potentiated the EC_3–10_ GABA currents upon α2β2 (213.3 ± 26.7%, *p* < 0.05, *n* = 3, Figure 4a), α3β2 (183.2 ± 21.1%, *p* < 0.05, *n* = 5, Figure 4b) and α5β2 (316.2 ± 71.5%, *p* < 0.01, *n* = 6, Figure 4d) receptors, the potentiation was not significantly reduced by flumazenil (200 μmol/L). However, the α4β2 receptor (Figure 4c) could not be effectively modulated by 200 μmol/L diazepam.

The α1β3 receptor was also tested in comparison to α1β2 receptor, in order to study whether the diazepam effect depend on β subunit present in the receptors. Unlike the β2-containing receptors, α1β3 (Figure 4e) receptor was insensitive to diazepam in the present study.

### 3.4. Effects of Diazepam on Some Cortical Neurons Could Not be Fully Antagonized by Flumazenil

**Figure 5:**
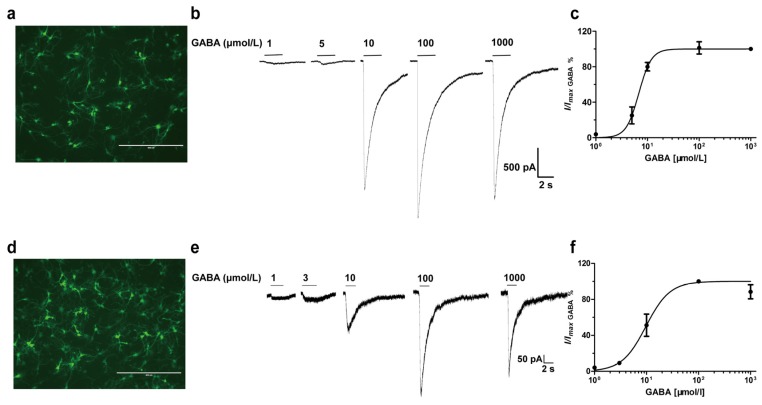
GABA concentration-relationship on hippocampal and cortical neurons. GABA (1–1000 μmol/L) was applied to acutely dissociated hippocampal or cortical neurons at 5 min intervals. (**a**,**d**) Immunofluorescence staining of hippocampal (**a**) and cortical neurons (**d**) with 160 kD neurofilament medium markers. (**b**,**e**) The current traces of different GABA concentrations from single cortical and hippocampal neuron. (**c**,**f**) GABA concentration-response curves in hippocampal and cortical neurons. Each point represents the normalized mean peak currents from 3 to 4 cells.

Hippocampal and cortical neurons were cultured for 7–14 days before electrophysiological tests (Figure 5a,d). GABA activated hippocampal (Figure 5b,c) and cortical (Figure 5e,f) neurons in a concentration-dependent manner, with EC_50_ values of 7.51 and 5.05 μmol/L, respectively.

**Figure 6:**
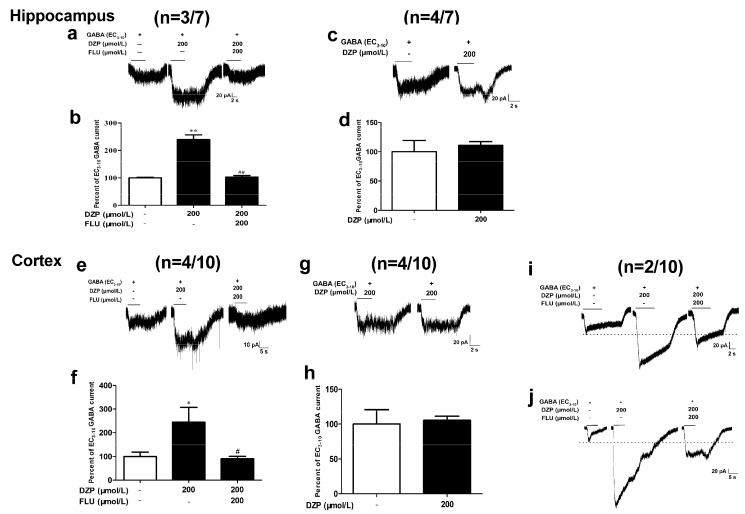
Modulatory effects of diazepam on hippocampal and cortical neurons. Diazepam (200 μmol/L) was co-applied with GABA (EC_3-10_, 1 μmol/L for hippocampal and 3 μmol/L for cortical neurons) to acutely dissociated neurons. Diazepam potentiate GABA currents on some hippocampal (3/7, **a**,**b**) and cortical (4/10, **e**,**f**) neurons and flumazenil (200 μmol/L) antagonized the effects. (4/7) hippocampal (**c**,**d**) and (4/10) cortical (**g**,**h**) neurons were insensitive to diazepam. (**i**,**j**) Current traces from two cortical neurons (2/10) showed the partial antagonism of flumazenil on diazepam. One-way ANOVA with Dunnett’s post hoc tests; * *p* < 0.05, vs. control group, ^#^ *p* < 0.05, vs. DZP group; *n* = 3–4.

Neurons showed distinct responses to diazepam and flumazenil. In the seven hippocampal neurons recorded, diazepam (200 μmol/L) potentiated GABA EC_3–10_ currents to 239.4 ± 17.4% (*p* < 0.01, *n* = 3) analyzed on (3/7) neurons, flumazenil (200 μmol/L) completely antagonized the effects of diazepam (*p* < 0.01, Figure 6a,b). However, the other (4/7) neurons were insensitive to 200 μmol/L diazepam (Figure 6c,d). Similar to hippocampal neurons tested, (4/10) cortical neurons were modulated by 200 μmol/L diazepam (244.5 ± 62.6%, *p* < 0.05, *n* = 4) and the diazepam effects was completely antagonized by 200 μmol/L flumazenil (*p* < 0.05, Figure 6e,f), (4/10) cortical neurons were insensitive to diazepam (Figure 6g,h). However, flumazenil only partially antagonized diazepam effects on (2/10) neurons. The potentiation of diazepam (200 μmol/L) on GABA EC_3–10_ currents was 302% and 551%, respectively, and flumazenil (200 μmol/L) decreased the elicited current to 168% and 207% (Figure 6i,j).

## 4. Discussion

Binary αβ GABA_A_ receptors are gaining increasing attention as the receptors have been reported to be expressed in the mammalian central nervous system [8,27] and certain benzodiazepines have been demonstrated to modulate some of these receptors [17,18,19,20,21]. Although functional properties of some αβ receptors have been reported [23,24,25,26,27], the variations in expression systems (Xenopus oocytes, HEK293 cells or Sf9 cells) and the methods of transfection and drug application make it difficult to compare the results from different studies. The present study was designed to study in detail the selectivity profile of benzodiazepines on αβ receptors and six receptor subtypes, including α1β2, α2β2, α3β2, α4β2, α5β2 and α1β3, were investigated. All receptor subtypes were expressed in the HEK293 cells using identical methods so as to enable more accurate comparisons between distinct receptor subtypes.

Since the αβ receptors do not contain the γ subunit, which is vital for the formation of the classical benzodiazepine binding site (α+/γ− interface), it can be supposed that the diazepam potentiation of GABA current on these receptors are mediated via a non-classical mechanism. The failure of flumazenil, the benzodiazepine antagonist at classical binding site, in antagonizing the diazepam effects also supports this supposition. The findings of the present study suggested that α1β2, α2β2, α3β2 and α5β2, but not α4β2, receptors may contain non-classical benzodiazepine binding sites. Although these novel binding sites are apparently different from the classical binding sites on αβγ receptors, the non-classical benzodiazepine modulation of αβ receptors showed similar dependence on α subunit with that of αβγ receptors (the α1-, α2-, α3- and α5- but not α4-containing receptors were sensitive to diazepam) [28,29], indicating that the classical and non-classical benzodiazepine binding sites may share some common structural basis on the α subunit. Desensitization of αβ receptors might affect diazepam effects during drug application (3–6 s), although desensitized effects of diazepam on αβ receptors remains unknown.

Three different non-classical diazepam binding sites, which are located at α+/β− [19], β2+/γ2− [30] (only in the β2γ2 receptor) interfaces and transmembrane domain [16], respectively, have been proposed on certain GABA_A_ receptors. Binary αβ receptors may expressed in 2α:3β or 3α:2β stoichiometry (Figure 1d) [19,31,32]. The present study used a α/β cDNA ratio of 1:1 to favor the formation of the 2α:3β instead of 3α:2β stoichiometry, and the diazepam effects on α1β3 in the present study are similar to that in the previous study [18]. Thus, in theory two β+/α− interface (GABA binding sites), two α+/β− interface, one β+/β− interface and the transmembrane domain were contained in αβ (2α:3β) receptors; therefore, diazepam was likely to bind to the α+/β− interface or the transmembrane domain (or both). Moreover, any GABA_A_ receptors (including αβγ receptors) containing the α+/β− interface or the transmembrane domain may be modulated through non-classical binding sites.

The structure of classical benzodiazepines, such as diazepam and midazolam, contain a fusion of a benzene ring and a diazepine ring [33,34]. Various subsequently-synthesized classical benzodiazepine site ligands (such as zolpidem), however, have non-benzodiazepine structures [35,36]. As for the classical benzodiazepine binding sites, partially distinguishable interaction with the binding pocket has been demonstrated in zolpidem and the classical benzodiazepines [37]. In addition, Ahmad Tarmizi et al. reported that zolpidem, but not diazepam, was able to bind to the α+/α− interface of α1β3 receptor (3α:2β) [19]. Here, we further showed the different effects of zolpidem compared to those of diazepam and midazolam on α1β2 receptor (2α:3β), indicating distinct binding potency for the α+/β− interface or the transmembrane domain among these three drugs. The differences in chemical structures may underlie the difference in the interaction of diazepam, midazolam and zolpidem to both the classical and non-classical binding sites in GABA_A_ receptors. This may contribute to the different pharmacological profile of imidazopyridine zolpidem compared to those of classical benzodiazepines [38].

Several studies proposed the non-classical benzodiazepine binding sites in certain recombinant GABA_A_ receptor subtypes [15,17,18,19]. In addition, diazepam was reported to produce flumazenil-insensitive inhibition of action potential firing in neocortical neurons [39]. In the present study, the flumazenil-insensitive effects were observed on some cortical neurons but not on hippocampal neurons, indicating that non-classical benzodiazepine binding sites exist in some cortical neurons. Although the present findings did not prove the existence of αβ receptors in vivo, contrasting responses of cortical and hippocampal neurons to diazepam and flumazenil may suggest the heterogeneity of GABA_A_ receptors within different brain regions, which is likely contribute to the diversity of inhibitory transmission within the cortex and hippocampus.

In conclusion, our findings demonstrate the flumazenil-insensitive benzodiazepine effects on αβ receptors, suggesting that the flumazenil-insensitive effects depend on both the receptor subtypes and the benzodiazepine structures. We also observed the non-classical benzodiazepine effects on cortical neurons. Further efforts are needed to elucidate the exact binding sites interacting with benzodiazepines on GABA_A_ receptors, and to explore the site-mediate behavioral effects in vivo. These will further enhance our understanding of the pharmacology of benzodiazepines, contributing to development of subtype- and binding site- specific drugs and separating pleiotropic effects of benzodiazepines.

## Figures and Tables

**Table 1:** Effects of GABA and benzodiazepines on αβ receptors.

Receptor	cDNA Ratio	GABA (μmol/L)		Hill Coefficient	Potentiation of GABA Currents (%)			*n*
		EC_50_	EC_3–10_		(Diazepam)	(Midazolam)	(Zolpidem)	
α1β2	1:1	1.23 (0.65–2.33)	0.1	0.62 ± 0.12	214.0 ± 31.4 *	171.8 ± 13.8 *	118.5 ± 7.0	3–5
α2β2	1:1	7.05 (5.16–9.63)	1.0	1.13 ± 0.19	213.3 ± 26.7 *	--	--	3–4
α3β2	1:1	7.94 (5.82–10.84)	0.5	0.96 ± 0.12	183.2 ± 21.1 **	--	--	3–5
α4β2	1:1	0.38 (0.32–0.44)	0.01	1.51 ± 0.16	87.4 ± 4.3	--	--	4
α5β2	1:1	7.13 (5.16–9.83)	0.5	0.98 ± 0.13	316.2 ± 75.2 **	--	--	3–6
α1β3	1:1	1.13 (0.63–2.00)	0.1	0.78 ± 0.15	107.2 ± 5.8	--	--	3

Primary parameters obtained from Figure 1, Figure 2 and Figure 3. Values are mean ± SEM; * *p* < 0.05, ** *p* < 0.01 vs. control group.
